# Isolation and Characterization of the Vascular Endothelial Growth Factor Receptor Targeting ScFv Antibody Fragments Derived from Phage Display Technology

**DOI:** 10.1021/acsomega.3c10158

**Published:** 2024-05-06

**Authors:** Hamid Kazemzadeh, Mahsima Bagheri, Maryam Sepehri, Elham Ebrahimi, Huan Wang, Shozeb Haider, Mitra Kheirabadi, Mohammad Reza Tohidkia

**Affiliations:** † Research Center for Pharmaceutical Nanotechnology, Tabriz University of Medical Sciences, Tabriz 51368, Iran; ‡ Basic Science Department, Faculty of Biology, Hakim Sabzevari University, P.O. Box 96179-76487, Sabzevar 571, Iran; § School of Pharmacy, University College London, London WC1N 1AX, U.K.

## Abstract

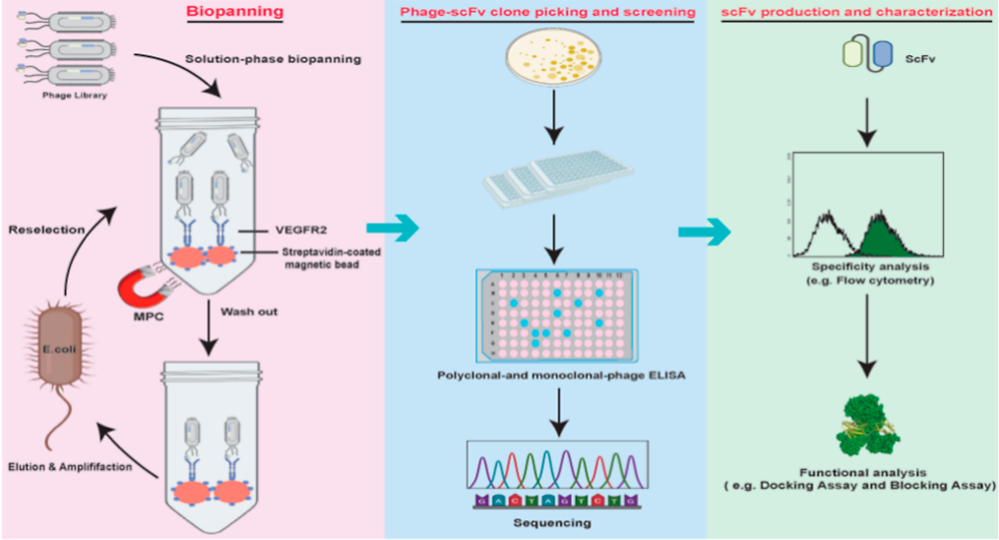

Angiogenesis, as a tumor hallmark, plays an important role in the growth and development of the tumor vasculature system. There is a huge amount of evidence suggesting that the vascular endothelial growth factor receptor (VEGFR-2)/VEGF-A axis is one of the main contributors to tumor angiogenesis and metastasis. Thus, inhibition of the VEGFR-2 signaling pathway by anti-VEGFR-2 mAb can retard tumor growth. In this study, we employ phage display technology and solution-phase biopanning (SPB) to isolate specific single-chain variable fragments (scFvs) against VEGFR-2 and report on the receptor binding characteristics of the candidate scFvs A semisynthetic phage antibody library to isolate anti-VEGFR-2 scFvs through an SPB performed with decreasing concentrations of the VEGFR-2-His tag and VEGFR-2-biotin. After successful expression and purification, the specificity of the selected scFv clones was further analyzed by enzyme-linked immunosorbent assay (ELISA), flow cytometry, and immunoblotting. The competition assay was undertaken to identify the VEGFR-2 receptor-blocking properties of the scFvs. Furthermore, the molecular binding characteristics of candidate scFvs were extensively studied by peptide–protein docking. Polyclonal ELISA analysis subsequent to four rounds of biopanning showed a significant enrichment of VEGFR-2-specific phage clones by increasing positive signals from the first round toward the fourth round of selection. The individual VEGFR-2-reactive scFv phage clones were identified by monoclonal phage ELISA. The sequence analysis and complementarity-determining region alignment identified the four unique anti-VEGFR-2-scFv clones. The soluble and purified scFvs displayed binding activity against soluble and cell-associated forms of VEGFR-2 protein in the ELISA and flow cytometry assays. Based on the inference from the molecular docking results, scFvs D3, E1, H1, and E9 recognized domains 2 and 3 on the VEGFR-2 protein and displayed competition with VEGF-A for binding to VEGFR-2. The competition assay confirmed that scFvs H1 and D3 can block the VEGFR-2/VEGF-A interaction. In conclusion, we identified novel VEGFR-2-blocking scFvs that perhaps exhibit the potential for angiogenesis inhibition in VEGFR-2-overexpressed tumor cells.

## Introduction

Angiogenesis is a multistep process associated with the formation, proliferation, and maturation of new blood vessels from the pre-existing vasculature.^1^ This process occurs under a variety of physiological conditions (e.g., embryonic development, tissue regeneration, wound healing, and menstrual cycle) or pathological states such as diabetic retinopathy, tumor metastasis, and survival, all of which are mediated by a plethora of vascular growth factors and their receptors.^2−4^ Compelling evidence suggests that the vascular endothelial growth factor receptor (VEGFR-2)/VEGF-A signaling pathway plays a critical role in promoting tumor angiogenesis and thereby tumor development and growth.^5,6^ VEGF-A is overexpressed in various types of human tumors, and the expression levels are associated directly with cancer prognosis.^7^ Consequently, targeting the VEGFR-2/VEGF-A axis by recombinant antibody fragments (e.g., Fab, fragment antigen binding, or scFv, single-chain variable fragments) or mAbs has been considered as a favorable strategy in the treatment of cancer by inhibiting tumor angiogenesis by inhibiting tumor angiogenesis.^8^ Antibody phage display, as an effective and powerful procedure, allows rapid and simple identification of fully human therapeutic mAbs with high affinity against an unlimited range of biological and nonbiological targets.^9^

In principle, the antibody phage display technique is based on coupling phenotype to genotype, in which the gene libraries encoding antibody fragments (scFv or Fab) are cloned next to gene III-encoded minor coat protein (pIII) of M13 filamentous bacteriophages to produce the phage particles displaying gene III fusion proteins on the phage surface.^10,11^ By applying a process known as “panning”, specific phage antibodies were isolated from a combinatorial antibody library via incubation with any given target of interest.^12^ Ramucirumab (cyramza), an anti-VEGFR-2 human IgG1, was approved by the US Food and Drug Administration for the treatment of metastatic gastric cancer in 2014.^13^ A phage displayed a human Fab library (Dyax) with 3.7 × 10^1^° independent clones served as the initial point for ramucirumab development. The affinity maturation of the primary selected clones (D2C6, D2H2, and D1H4) with nanomolar affinity resulted in the Fab clone 1121 (IMC-1121B), showing an over 30-fold increase in VEGFR2-binding. This clone was further engineered into the human intact IgG1 version, ramucirumab, reaching an impressive affinity of 50 pM.^14,15^ Furthermore, a number of mAbs directed at VEGFR-2, including olinvacimab (Phase II, glioblastoma), pulocimab (Phase I, solid tumors and Phase I/II, GE Junction cancer), and vulinacimab (Phase I, solid tumors), which are in different stages of clinical trials, underscore the therapeutic potential of anti-VEGFR2 mAbs^16^ (IMGT/mAb-DB).

Among the various types of antibody fragments, scFvs are the most commonly used for generating phage display libraries.^17^ scFv with a molecular weight of approximately 28 kDa encompasses the variable regions of the heavy (VH) and the light (VL) chains connected via a 15 amino acid linker polypeptide; three hypervariable regions in the VH (H1, H2, and H3) and VL domains (L1, L2, and L3) are located at the antigen binding sites, which are known as complementary determinant regions (complementarity-determining regions (CDRs)).^18,19^ The advantages of these antibodies in contrast to whole mAbs are smaller size, greater and uniform permeability in tumor sites, less toxicity and immunogenicity due to the rapid bloodstream clearance and the lack of Fc region, and high-yield and cost-effective production in bacterial systems.^20^

Biopanning is the main stage in the isolation of antibodies from phage libraries, where a phage library is incubated with a target antigen. Nonspecific phages are removed after several stages of washing, following which the specific phages are separated from the target.^21^ In general, alternative methods have been employed for biopanning, including solid-phase panning, soluble-phase panning by means of biotinylated antigens, guided selection using mouse monoclonal antibodies, whole-cell panning, and magnetic sorting techniques.^22,23^ Although selection in the solid-phase is the most commonly used method for the isolation of antibodies using phage display technology, this method is sometimes accompanied by the isolation of low-affinity, nonspecific binders as well as binders not recognizing the native conformation of the target. Solution-phase biopanning (SPB) is considered a robust alternative that eliminates these problems as it assists in the enrichment of high-affinity phage binders against the native conformation of targets. In the present study, we applied SPB by using two different VEGFR-2 as the selection targets (His-tagged VEGFR-2 and biotinylated VEGFR-2) to ensure the isolation of the conformation-specific scFvs.

## Materials and Methods

### Library Preparation, Helper Phage, and Bacterial Strains

An artificial human Single Fold scFv Libraries I + J (Tomlinson I + J), which is composed of 1.47 × 10^8^ and 1.37 × 10^8^ scFv clones, respectively, KM13 helper phage, TG1-Tr, and HB2151 *E. coli* strain, was purchased from Source Bioscience (Nottingham, UK). In this study, TG1 *E. coli* for scFv-phage propagation and HB2151 *E. coli* for soluble scFv production were utilized. Sequences encoding scFvs were cloned next to g3p into a phagemid vector, pIT2, with His- and c-Myc tags, which can be used for detection and purification by chromatography. The library rescue and helper phage preparation were performed according to the instruction manual of the Tomlinson phage antibody library available online.^24^

### Biopanning

In the first step, recombinant phage scFvs (4.5 × 10^14^ cfu/ml) were rescued by the KM13 helper phage from library I and subjected to affinity selection by four rounds of SPB. The recombinant phage antibodies and magnetic bead His-tag (Clontech, Takara Bio) or Dynabead-streptavidin Myone-T1 (Thermo Fisher Scientific, Waltham, MA) were incubated with blocking buffer and 3% BSA (phosphate-buffered saline containing 0.05% Tween-20 and 3% bovine serum albumin) for 90 min. The panning was performed with decreasing concentrations of two types of the VEGFR-2 antigen: VEGFR-2-His tag (SinoBiological-10012-H08H) in rounds 1 (100 nM) and 2 (50 nM) and the VEGFR-2-biotin (Sino Biological-10012-H08H-B) in rounds 3 and 4 (50 nM) on a rotator for 90 min. The phage–protein complexes were captured by adding the blocked magnetic bead His-tag or Dynabead-streptavidin Myone-T1 using an MPC. The nonspecifically bound phages were washed out by 4–6 times washing step with PBST (PBS containing 0.1% Tween-20). Next, specifically bound phages were eluted by incubation with 250 mM imidazole (for rounds 1 and 2) and 500 μL (for rounds 3 and 4) pancreas trypsin (1 mg/mL) (Sigma-Aldrich Co., Taufkirchen, Germany) for 15 min. Half of the eluent was amplified in TG1 *E. coli* to prepare for the next rounds of selection, and the other half was stored for polyclonal phage ELISA. To increase the work efficiency and eliminate bead- and streptavidin-reactive recombinant phages, the preabsorption step was performed for each round of selection by incubating the recombinant phages with magnetic bead His-tag and Dynabead-streptavidin.

### Polyclonal Phage ELISA

To determine the rate of enrichment and binding activity of phage-scFvs to VEGFR-2, ELISA was performed for amplified phages after four rounds of selection. The high-binding 96-well microtiter plates were coated with 3 μg/mL BSA-Biotin (Sigma-Aldrich Co., Taufkirchen, Germany) in PBS at 4 °C overnight, followed by streptavidin at a concentration of 2 μg/mL in PBS at RT for 90 min, in sequence to positive and negative wells. The positive wells were incubated with 1 μg/mL biotinylated VEGFR-2 at RT for 90 min; meanwhile, the negative wells were incubated for 60 min with blocking buffer, 2% MPBS (PBS containing 2% nonfat dry milk). After a 3-time washing step, all the negative and positive wells were blocked for 90 min at RT, and 100 μL of 2% MPBS contained the phages from each round of biopanning for 90 min at RT. To detect phage-scFv binding to a given antigen, a 1:3000 dilution of primary mouse anti-M13 monoclonal antibody (GE Healthcare, catalog #27-9420-01) and a 1:5000 dilution of secondary goat antimouse IgG-conjugated HRP (Invitrogen, catalog #M30107) were added to the plates at RT, each for 60 min. After each incubation step, the plate was washed four times with PBST. The plate was stained with 100 μL/well by TMB (tetramethylbenzidine) as an enzyme substrate, and the peroxidase reaction was stopped after 5–10 min by adding 5% sulfuric acid. The progression of the absorbance was read at optical density (OD) of 450 subtracted from 630 nm in a BioTeck ELISA reader.

### Monoclonal Phage ELISA

Monoclonal phage ELISA was performed at this stage to confirm the findings related to enrichment as well as to screen individual phage-scFv clone specific to VEGFR-2. For this purpose, randomly selected bacterial colonies on TYE-AG (100 μg/mL ampicillin and 4% glucose) plates were inoculated into 96-well cell culture plates containing 100 μL/well 2× YT-AG (100 μg/mL ampicillin and 4% glucose) and grown overnight at 37 °C with shaking. A 5 μL aliquot from the overnight cultures was transferred to 200 μL 2× YT-AG and incubated at 37 °C with shaking until OD_600_ of 0.4. Afterward, the culture was infected with the KM13 helper phage, and then, the culture medium was changed to 2× YT-AKG (100 μg/mL ampicillin, 50 μg/mL kanamycin, and 4% glucose) and incubated overnight at 30 °C with vigorous shaking vigorously. The supernatant of individual colonies was transferred to 96-well plates containing 4% MPBS to block phage-scFvs and then used for ELISA as described before in polyclonal phage ELISA.

### Clone Diversity and Sequence Analysis

Phagemid vectors from the ELISA positive scFv clones were extracted by a Bioneer kit (Bioneer, Takapou Zist, Tehran, Iran). The antibody sequences in the plasmid were amplified by two primers pHEN seq (5′-CAG GAA ACA GCT ATG AC-3′) and LMB3 (5′- CTA TGC GGC CCC ATT CA-3′) to verify full-length scFv inserts by agarose gel electrophoresis. DNA sequencing was done by the Takapou Zist Company using the LMB3 primer, and then, the sequence diversity was analyzed by Chromas software (Technelysium Pty Ltd., Queensland, and Australia). Finally, sequence diversity was accomplished by alignment of CDR regions using the VBASE2 database.

### Expression and Purification of Soluble scFvs

To express soluble scFvs, the ELISA-positive phage-scFv clones with unique CDRs were transformed into the *E. coli* HB2151 strain. Then, individual HB2151 scFv clones were grown in 200 mL of 2× YT–AG at 37 °C until OD_600_ of 0.9. The bacteria pellet was harvested by centrifugation at 3000*g*, RT for 10 min and resuspended in 2× YT-A containing 0.4 M sucrose and 1 mM IPTG to induce soluble antibody expression at 30 °C for 5 h. To extract antibody fragments from periplasmic space, the bacterial culture was harvested and resuspended in 1/20 original culture volume of ice-cold TES buffer (50 mM Tris pH 8, 1 mM EDTA, and 20% (w/v) sucrose). Following the centrifugation at 20,000*g* for 30 min at 4 °C, the periplasmic fraction containing soluble scFvs were collected and then dialyzed by a cellulose membrane with 12 kDa cutoff against PBS buffer overnight at 4 °C.^25^

The purification process of His-tagged soluble scFvs was established by immobilized metal affinity chromatography resin using TALON Ni-NTA agarose (Clontech Laboratories, Takara Bio USA) according to the manufacturer’s protocol. The resin-captured scFvs were eluted by 150 mM imidazole and dialyzed against PBS by Maxi Pur-A-Lyzer Dialysis tubes with 12 kDa cutoff (Sigma-Aldrich Co.).

### SDS-PAGE and Western Blotting

Evaluation of expression and purification were done by 12% sodium dodecyl sulfate–polyacrylamide gel electrophoresis (SDS-PAGE) and immunoblotting according to the instructions of Bio-Read Company (Mini Protean Tetra Cell and the semidry Trans-Blot system). After separating protein bands by SDS-PAGE, one gel was immersed in Coomassie brilliant blue G-250 and the other one was blotted onto nitrocellulose membrane followed by blocking with 5% skim milk overnight at 4 °C. For staining of antibody fragments, the primary anti-*c*-Myc antibody with dilutions of 1:3000 and secondary goat antimouse IgG conjugated with HRP at a dilution of the 1:5000 was used in sequential on the membranes. Finally, to promote visualization of protein bands, an X-ray film was used subsequent to electrochemiluminescence (ECL) solution addition onto the membranes.

### Soluble scFvs ELISA

To investigate the binding activity of the purified antibody clones, soluble scFv ELISA analysis was used. For this purpose, the purified scFvs at 1 μg/mL were applied for ELISA assay, similar to monoclonal ELISA, except that HRP-conjugated protein A was used instead of the anti-M13 antibody.^26^

### Cell Culture and Flow Cytometry

Flow cytometry analysis was performed to verify specific binding of scFvs to the native structure of VEGFR-2 expressed on the cell surface based on our previous work.^21,27^ HEK-293 and HEK-293-KDR as VEGFR-2-negative and -positive cell lines were grown in Dulbecco’s modified Eagle’s medium/F12 and Roswell Park Memorial institute media, respectively, supplemented with 10% fetal bovine serum. The cells were cultured to 90% confluence at 37 °C in a humidifying incubator with a 5% CO_2_ atmosphere. The culture medium was renewed every 2 or 3 days, and the cells were harvested with a 0.25% trypsin–EDTA solution with centrifugation at 250*g*, 4 °C for 10 min. For flow cytometry, 250,000 cells were collected for each sample and were blocked with FACS buffer (PBS containing 1% BSA and 0.03% NaN_3_). The cells were stained with 10 μg of scFvs in FACS buffer for 60 min on ice. Following the washing step, the cells were sequentially incubated with 0.5 μg/sample of anti-*c*-Myc antibody (Santa Cruz Biotechnology-sc40) for 60 min and 1 μg/sample of FITC-labeled antimouse IgG (Biolegend-406001) in the dark for 30–45 min on ice. Finally, the cells were resuspended in PBS and analyzed using a FACSCalibur flow cytometer (Becton Dickinson, Franklin Lakes, NJ, USA) for the binding behavior of scFvs. After each incubation, all wash steps were carried out with FACS buffer and centrifuged at 250*g*, 4 °C for 10 min.

## Competitive ELISA Assay

Competitive binding of scFvs was assessed by indirect ELISA. A mixture of varying concentrations of scFvs (10–250 nM) with 100 ng of biotinylated recombinant human VEGFR-2 was incubated at RT for 1 h.^3^ Next, the mixture was transferred to 96-well plates which were coated with 200 ng/well of VEGF-A (BioBasic-RC216-16) at 4 °C overnight. After blocking by MPBS and washing 3 times with PBS-T, the plates were incubated with 100 μL of streptavidin-HRP antibody (R&D Systems-890803) for 1 h at RT. Following the washing step, development of color was carried out by adding TMB substrate solution, and then the enzymatic reaction was stopped by 5% H_2_SO_4_. The OD was determined at 450 nm subtracted from 630 nm (background absorbance) by using a BioTeck ELISA reader.

### Homology Modeling

Three-dimensional (3D) structure of the scFvs was modeled using the Swiss Model server (https://swissmodel.expasy.org/). One template with maximum sequence identity and query coverage was used for structure prediction (Template 1: PDB code: 5GS3-A). The model refinement was performed through a short molecular dynamics simulation to relieve any steric clashes using the Galaxy Web Server (https://galaxy.seoklab.org).^28,29^ The final model was validated using the SAVES (https://saves.mbi.ucla.edu), PROSA (https://zlab.umassmed.edu/bu/rama/), and QMEAN (https://swissmodel.expasy.org/qmean) servers.

### Molecular Docking

#### scFvs-VEGFR-2 Docking

Molecular docking was performed between domain 2 and 3 VGFR2 (PDB code: 2X1X) and scFvs using the ROSETTA docking server (http://rosie.rosettacommons.org), a comprehensive web-based program for predicting the structure and interactions of macromolecules.

The program searches for side chains that have the least free energy for antibody–protein interactions by performing docking and side-chain compatibility. The output file consists of the top 10 complexes that are classified based on total energy scores and interface energy. LIGPLOT 1.4.5 (https://www.ebi.ac.uk/thornton-srv/software/LIGPLOT) and UCSF Chimera 1.11.2 (https://www.cgl.ucsf.edu/chimera/) were used to analyze the complexes.

## Results and Discussion

### Polyclonal Phage ELISA to Monitor

**Figure 1:**
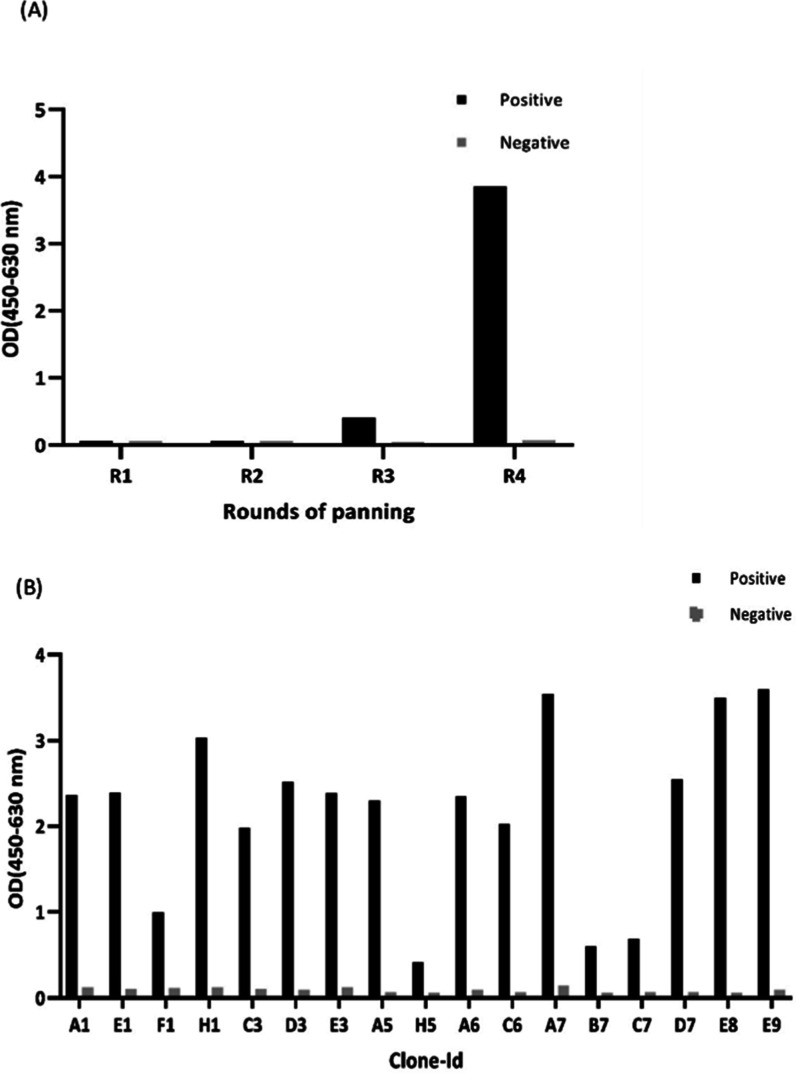
(A) Polyclonal phage ELISA assay. The recombinant phages amplified after each round of panning were incubated in wells containing VEGFR-2 as the selection target (positive), which are coated via biotinylated-BSA and streptavidin, and with wells lacking the target antigen (negative). The binding ability of recombinant phages to given target was measured by the primary mouse anti-M13 mAb and secondary goat antimouse IgG-conjugated HRP. The optical values (OD) were read at 450 nm subtracted from those at 630 nm. (B) Monoclonal phage ELISA. The individual phage-scFv clones were screened for specific binding to VEGFR-2 by ELISA. The phage-scFvs were produced in the supernatant of 96-well cell culture microplates and then applied to positive ELISA plates (coated with the target antigen) and negative control plates (coated with biotinylated-BSA and streptavidin without the target antigen). Detection of phage binders was the same as the polyclonal phage ELISA. The optical values (OD) were read at 450 nm subtracted from 630 nm.

For affinity selection of recombinant phages, biopanning steps were performed in four sequential rounds accompanied by a predepletion step to eliminate the nonspecific binder in favor of enrichment of specific phage-scFv clones. The amplified phages, after each round of panning, were added to the antigen-coated wells (positive) and the antigen-free wells (negative control). There is a significant increase in the OD value of specific phages after the third round of panning, indicating high enrichment of antigen-specific phage-scFvs in the round of 4 (Figure 1A).

### Screening Individual Specific Phage-scFvs and Sequence Diversity

Monoclonal phage ELISA was performed for screening of individual scFv clones after round 4. The phage-scFvs with a 3-fold ELISA OD over the background signal were selected as positive clones; 16 out 75 analyzed scFv clones showed specific binding to the VEGFR-2 proteins (Figure 1B). The presence of the full-length gene encoding scFvs and sequence diversity of the ELISA-positive phage clones were analyzed by PCR and DNA sequencing, respectively. According to PCR findings, 9 scFvs (A7, E3, C3, C6, E1, H1, A1, D3, and E9) were shown to harbor the full-length scFv insert. Based on the VBASE2 database, the sequence analysis and alignment of CDR regions identified the three scFvs H1, C6, and C3, as well as two scFvs E1 and A1 are identical, whereas scFvs E9, D3, and A7 displayed unique sequence (Table 1). ScFvs A7 and E3 were excluded from further analysis due to the presence of a stop codon (TAG) within their variable regions, rendering them incapable of expressing as soluble proteins. This exclusion can be attributed to the amber stop codon suppression capability of the TG1 strain that is utilized for propagation and production of recombinant phage-scFvs, while the nonsuppressor strain HB2151 is employed for expression and production of soluble scFvs devoid of the phage pIII.

**Table 1:** Alignment and Comparison of CDR-Sequence Regions of scFvs with the VBASE Database

selected phage scFvs	frequency (%)	VH			VL		
		CDR1	CDR2	CDR3	CDR1	CDR2	CDR3
E1	2/16 (12.5)	GFTFSSYA	INGAGSYT	AKYSGSFDY	QSISSY	AAS	QQNSTDPAT
H1	3/16 (18.8)	GFTFSSYA	ISGNGGYT	AKYSGSFDY	QSISSY	AAS	QQNAYSPAT
E9	1/16 (6.2)	GFTFSSYA	ISASGGYT	AKNSGSFDY	QSISSY	YAS	QQGGYAODT
D3	1/16 (6.2)	GFTFSSYA	ISASGGYT	AKTAKAFDY	QSISSY	DAS	QQNTTAPTT

### Production of Soluble scFvs

**Figure 2:**
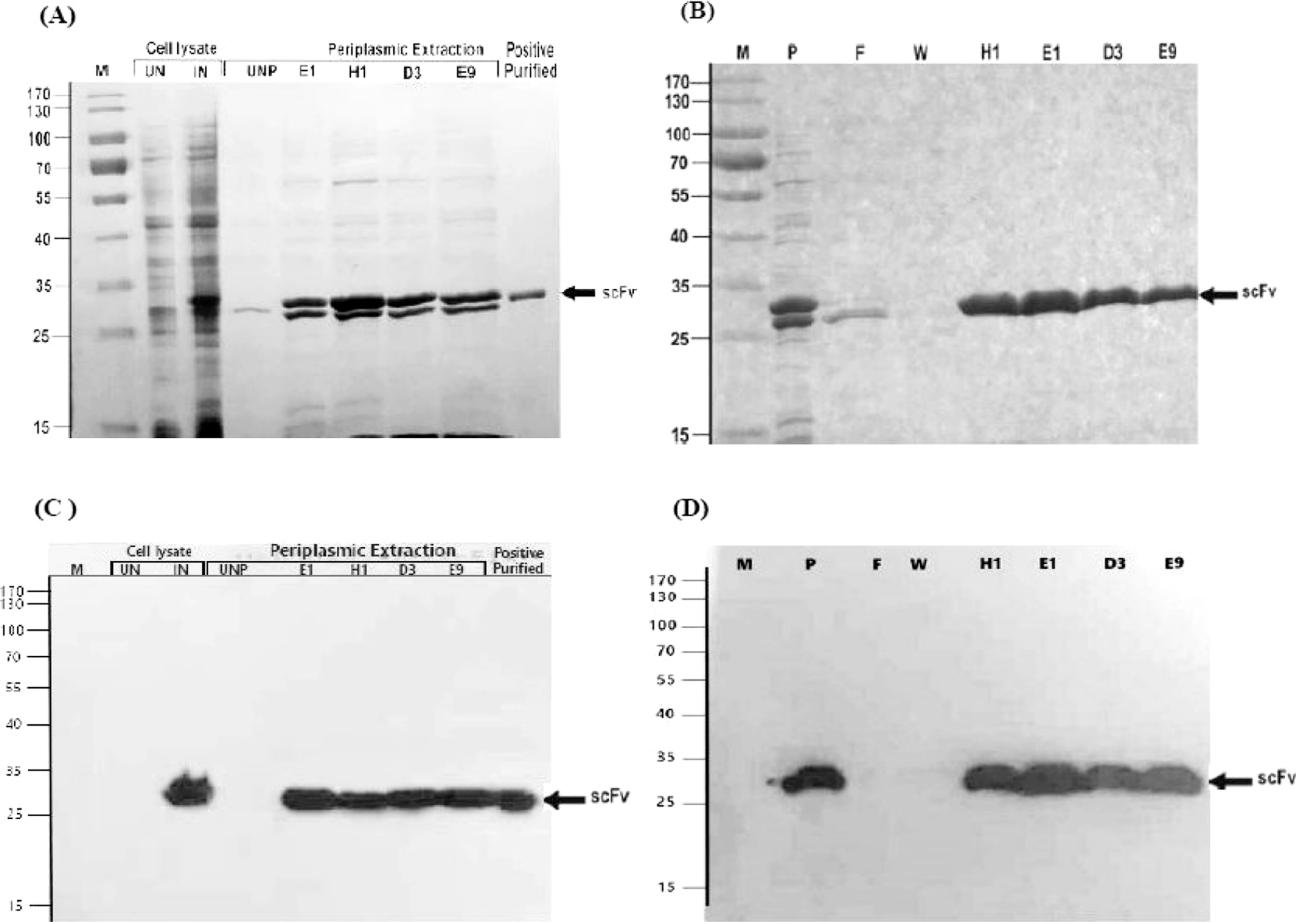
SDS-PAGE and Western blotting analysis. The expression and purification of the individual, unique clones were verified by 12% SDS-PAGE and blotting. Panels A and B depict the expression of soluble scFvs with an expected molecular size of 28 kDa. Panels C and D show immunoblotting of soluble scFvs with homogeneity and purity above 99%. The approximate molecular weight of scFvs is indicated by arrows. M, molecular weight standards in kDa; UN, uninduced cell lysates; IN, induced cell lysates; UNP, uninduced periplasmic extracts; induced periplasmic extracts related to the selected clones (i.e., E1, H1, D3, and E9); P, periplasmic extraction; F, flow-through; W, wash fraction; and purified scFvs (i.e., E1, H1, D3, and E9).

To obtain a sufficient amount, the soluble scFvs (i.e., H1, E9, E1, and D3) with high activity were expressed and purified. The SDS-PAGE and immunoblotting analyses of the expression and purification processes showed the successful production of soluble scFv proteins with 99% purity and a molecular weight of ∼28 kDa. This is consistent with the theoretical molecular weight of the scFvs calculated from the amino acid sequence of their constituent chains (Figure 2).

### Binding Specificity Analysis of the Purified scFvs ELISA

**Figure 3:**
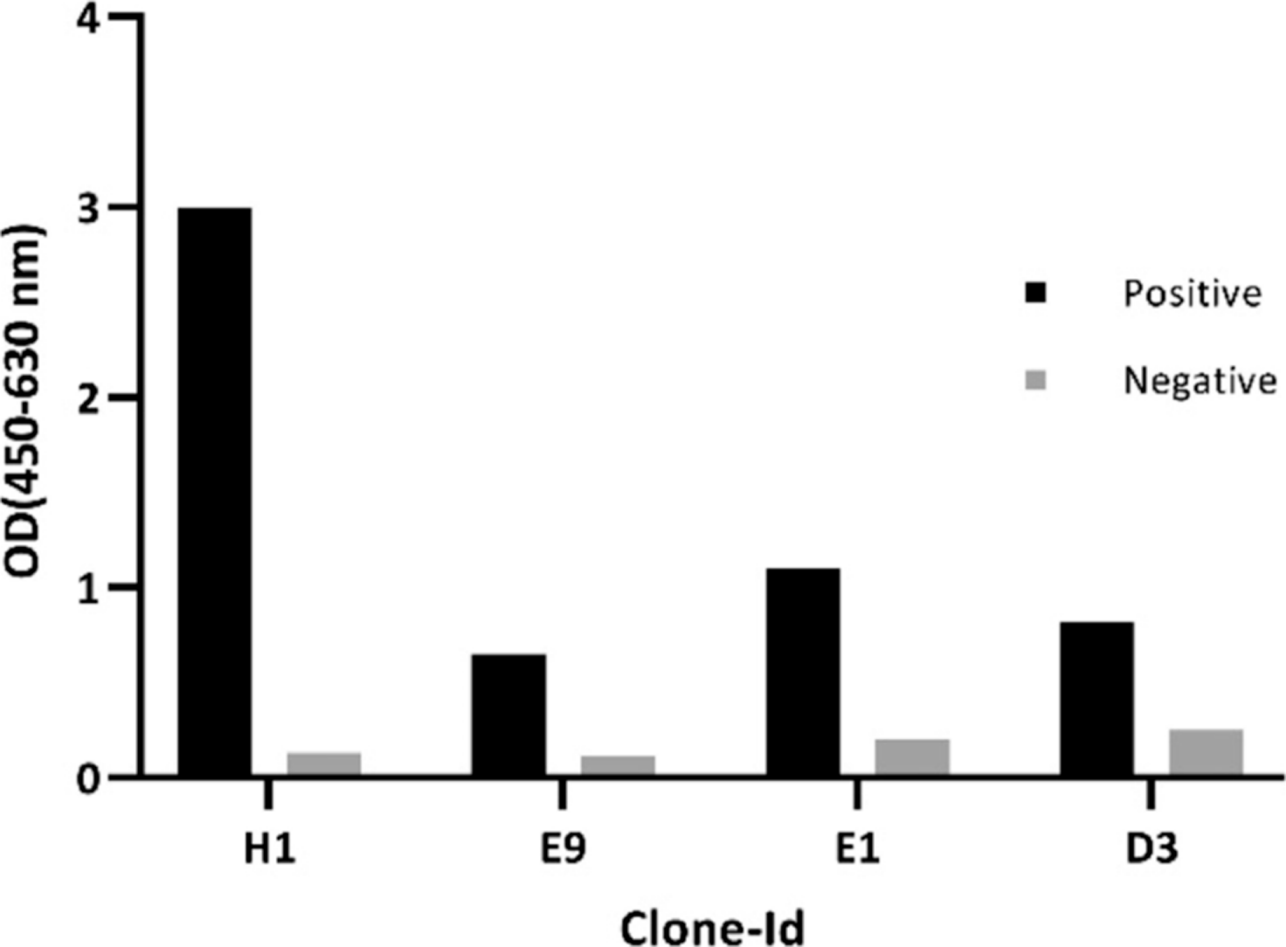
ELISA assay using purified scFvs The purified scFvs H1, E9, E1, and D3 were incubated with biotinylated VEGFR-2-coated wells (positive) and also wells lacking biotinylated VEGFR-2 (negative control). Anti-*c*-Myc mAb and HRP-conjugated goat antimouse antibodies were sequentially used to detect specific binding. The optical values (OD) were read at 450 nm subtracted from 630 nm.

To further characterize the binding specificity, the purified scFvs were again applied to an ELISA assay similar to that in monoclonal phage ELISA. Overall, the findings suggested that the 4 scFvs produced were specifically able to bind to VEGFR-2 (Figure 3).

### Flow Cytometry Analysis

**Figure 4:**
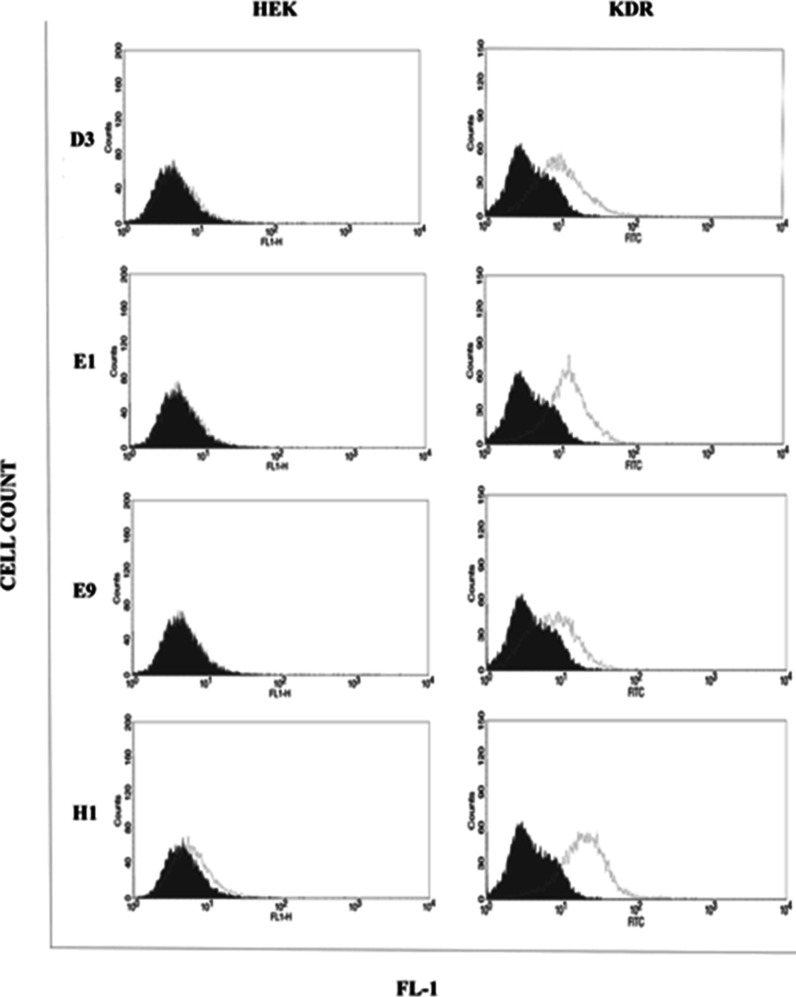
Cell binding activity by flow cytometry. The HEK and HEK-KDR cells as the VEGFR-2-negative and -positive cell lines, respectively, were stained with the scFvs. The specific cell binding of the scFvs was identified by sequential incubation with the anti-*c*-Myc antibody and FITC-labeled goat antimouse IgG. The *x*-axis and *y*-axis in each histogram plot illustrate fluorescence staining and cell counts, respectively. The filled gray color plots depict isotype control (without the scFv staining) for each cell line, and the empty green line plots are representative of the scFv staining. The viable cells (10,000 cells) were analyzed by FACSCaliburTM, excluding dead cells stained by propidium iodide.

To investigate the specific binding of the scFvs to the native structure of VEGFR-2 expressed on the cell surface, flow cytometry was performed. The cell binding activity of the scFvs was detected by an increase of fluorescent signal in VEGFR-2 positive cell line when compared with the VEGFR-2 negative cell. As shown in Figure 4, all the isolated scFvs appeared to have moderate (scFvs D3 and E9) to high (scFvs E1 and H1) binding capacity to the native structure of VEGFR-2 expressed on the HEK-KDR cell surface as a VEGFR-2-positive cell line. The flow cytometry results are consistent with those of the ELISA and docking analyses. None of the scFvs showed binding to HEK cells as a VEGFR-2-negative cell line.

### Blocking VEGF-A Binding Site on VEGFr-2

**Figure 5:**
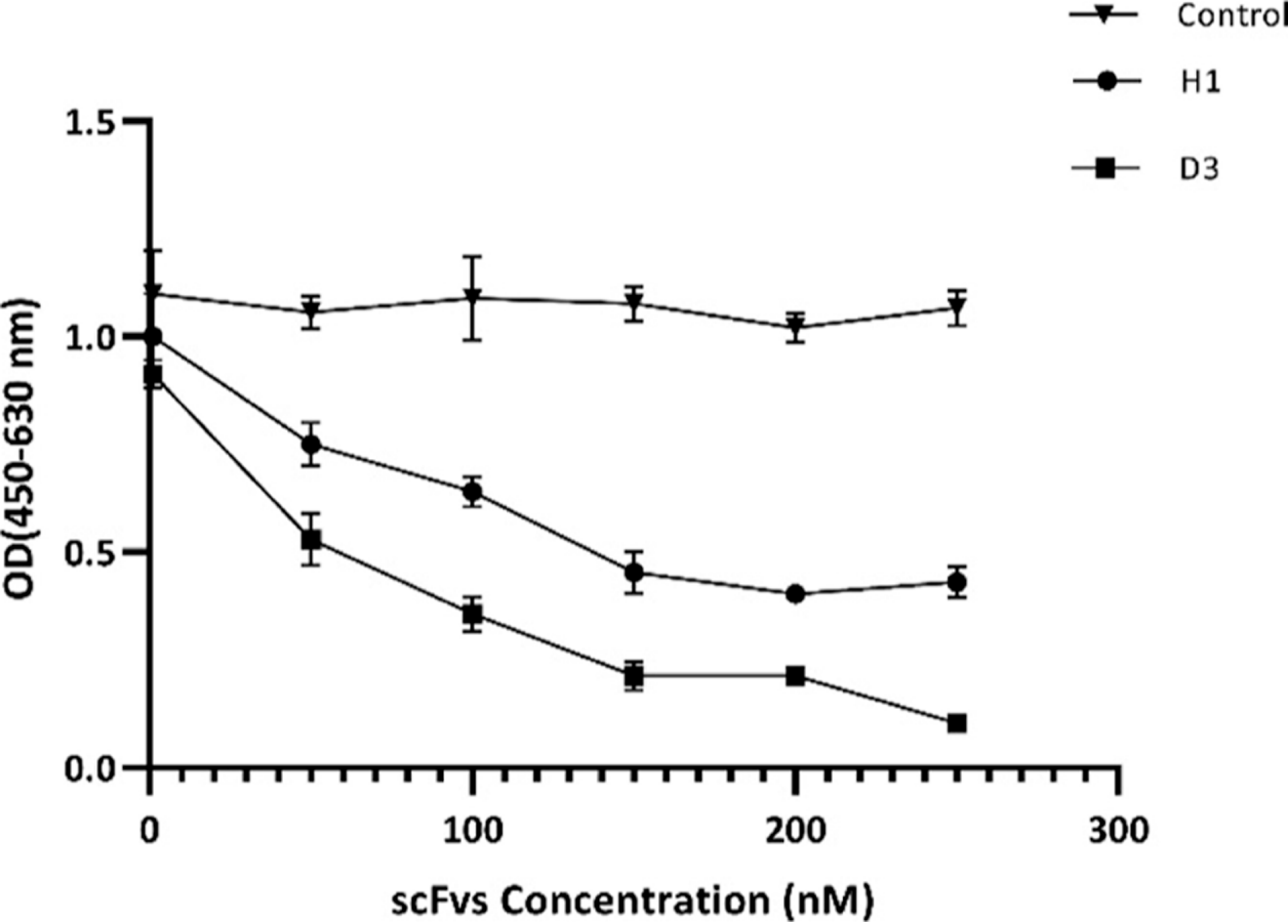
Competitive ELISA assay. A constant concentration of biotinylated VEGFR-2 in solution was incubated with various concentrations of two scFvs (H1 and D3) and a negative control anti-BSA before transferring to 96-well plates coated with VEGF-A. The binding of the biotinylated VEGFR-2 to VEGF-A was detected by streptavidin-HRP and TMB enzymatic substrate reagents. The optical values (OD) were read at 450 nm and subtracted from 630 nm.

To assess the functional binding characteristics of the purified soluble scFvs to VEGFR-2, we applied two VEGFR-2-specific scFvs (H1 and D3) and anti-BSA (as a negative control) for a competition assay. While scFv D3 displayed the maximum interference with VEGF-A binding to the recombinant VEGFR-2 with increasing concentration, scFv H1 represented binding to VEGFR-2 and blocking impact to some extent, in comparison with scFv D3. Additionally, anti-BSA scFv failed to exhibit an impulsive reaction (Figure 5). These results suggest that scFv D3 can effectively compete with VEGF-A for binding. On the other hand, scFv H1 also shows some binding and blocking activity.

### Bioinformatics Analysis

**Figure 6:**
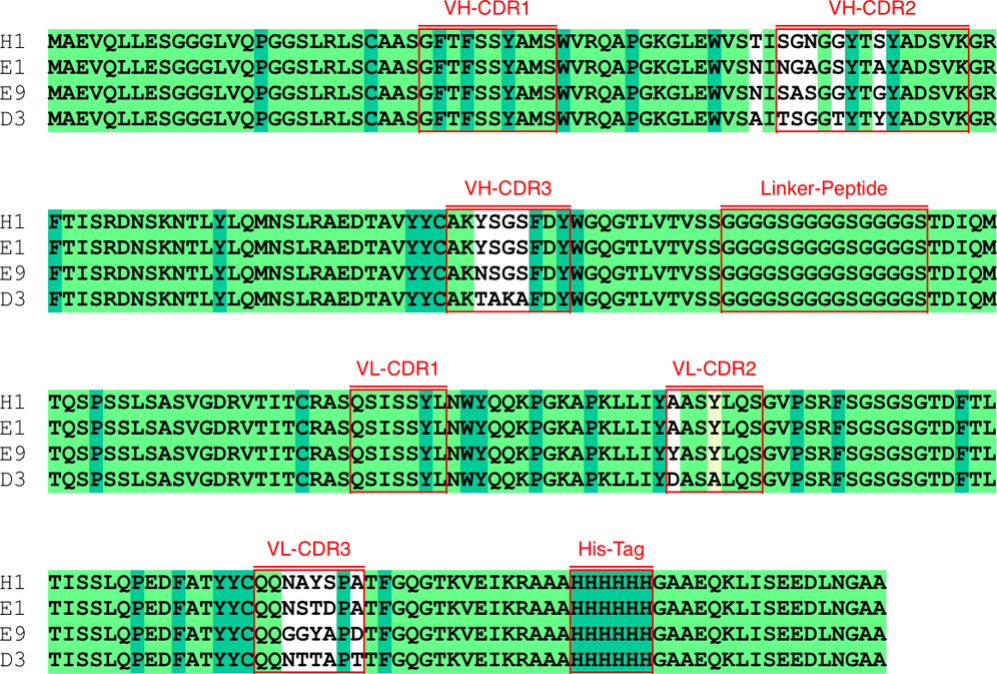
Alignment of primary sequences of antibodies (H1, E1, E9, and D3) with VH, VL, and linker CDR regions. * indicates the residues conserved in the four sequences.

To analyze the binding mode of interaction between scFvs and VEGRF2 in detail, the models of scFvs were first built and then docked to VEGFR-2. The primary structural analysis of scFvs fragments (E1, H1, E9, and D3) demonstrated that they have the same light (VL) and heavy (VH) chains and linkers except their CDRs (Figure 6).

### Homology Modeling

3D models of scFvs were built with homology modeling with the Swiss model using the template sequence (PDB code: 5GS3-A). The best model of scFv was selected based on Global Model Quality Estimate indices: for D3: Model1 was selected with GMQE:0.80; for model E1, Model2 was selected with GMQE:0.78; for E9 scFv, Model3 was selected with GMQE:0.80 and for H1; Model2 was selected with GMQE:0.73 (Table-S1). To select the best model for each scFv, models were evaluated using QMEAN, Procheck, and ProSA. The selected models were further refined using short bursts of molecular dynamics to relieve any steric clashes. The best model obtained for each scFvs was chosen for Ramachandran plot analysis (Figures S1–S4 and Table S2).

### Protein–Protein Docking

Molecular docking of antibodies modeled with the receptor protein was performed using Rosetta antibodies, and the top 10 complexes with the best docking scores were obtained for all four antibodies.

Rosetta categorizes the score for each complex based on two factors: total energy (total score) and interface energy (interface score). The total score is the total score that reports the total energy of the set, and the interface score, or I_SC, shows the interface score, which is calculated from the total set score minus the total score of each member separately. The I_SC factor is suitable for ranking the results of molecular docking. The best complex of ten complexes for all four antibody sequences (Table S3) was selected based on the two factors of total energy (total score) and interface energy (interface score) (Table 2).

**Table 2:** Best scFvs-VEGFR-2 Complex Determined by Rosetta Docking

H1		D3		E9		E1	
interface score (I_SC)	total score	interface score (I_SC)	total score	interface score (I_SC)	total score	interface score (I_SC)	total score
–4.138	–396.791	–3.589	–335.349	–4.025	–317.145	–5.285	–313.725

Hydrogen bonds formed between VEGFR-2 and the scFv molecules were predicted based on the intermolecular interaction analysis. For scFv D3, Gln161 in CDR3-VH and Tyr183 in CDR2-VH formed hydrogen bonds, respectively, with Gly193 and Ser191 and Gln13 (Figure S5). In the E1-VEGFR-2 complex (Figure S6), interactions hold between Gln161 in CDR1-VL and Asp138 in VEGFR-2; Ser226 and Asp228 in CDR3-VL and Asn155 and Arg156 in VEGFR-2, respectively; and Ser102 in CDR3-VH and His148 in VEGFR-2. ScFv H1 interacted with VEGFR-2 via one hydrogen bond formed between Asn56 in CDR2-VH and Asn140 in VEGFR-2 and two hydrogen bonds between Thr134 and Thr139 in regions outside CDRs and His14 and Ser9 in VEGFR-2 (Figure S7). ScFv-E9 just formed hydrophobic interactions through CDR1-VL, CDR2-VL, CDR2-VH, and Loop1 in CDR1-VH with VEGFR-2 (Figure S8).

**Figure 7:**
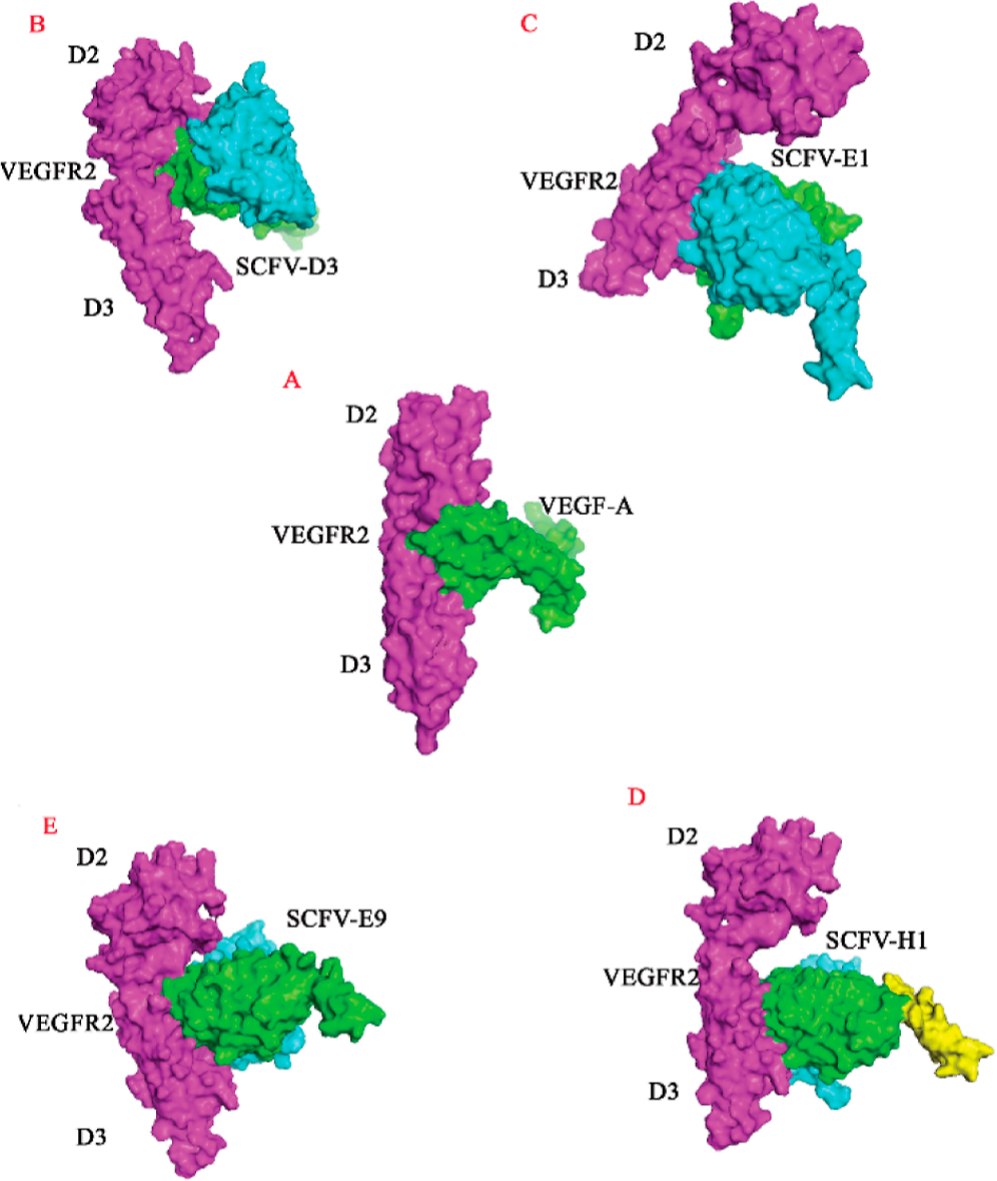
Schematic view of D2 and D3 domains of VEGFR-2 (purple color) in complex with heavy chain (green) and light chain (cyan) of (A) VEGF-A, (B) scFv-D3, (C) scFv-E1, (D) scFv-H1 (yellow linker), and (E) scFv-E9.

The analysis of the VEGF-A/VEGFR-2 complex (PDB code: 3V2A) showed that VEGF-A is located in the flexible cavity between the D2 and D3 domains of the extracellular domain of VEGFR-2 (Figure 7A). The docked analysis showed that the isolated scFvs (D3, E1, H1, and E9) can bind to a valley region located between the D2 and D3 domains. Among the scFvs, D3 and E9 occupied the same region on the VEGFR-2 as the VEGF-A and potentially compete with VEGF-A (Figure 7B–E).

## Discussion

The anti-VEGFR-2 scFvs that compete with VEGF binding to the extracellular domain of VEGFR-2 were discovered using a phage display library. Using a SPB shape in which decreasing concentrations of the target antigen were used in successive rounds of the selection, the antigen-specific phage-scFvs were enriched at round 4 (Figure 1A). The clone screening and sequencing results indicated that 21 percentage of scFv clones, possessing 4 unique acid nucleic sequences, could recognize the target (Table 1).

In contrast to the solid-phase selection which is accompanied by frequent isolation of the antibodies either not recognizing the native structure of protein expressed on cell surface or specific for solid support substances,^30−32^ the solution-phase method assists isolation of conformation-specific scFv clones. Furthermore, the solid-phase methodology due to introducing avidity effect during selection processes often is not able to enrich antibody fragments with higher biding affinity, while the solution-phase provides the selection of rare binders with high affinity via avoiding avidity effect as well as controlling antigen concentration during each round of selection.^33,34^ Therefore, to obtain high affinity scFv antibodies, we designed a solution-phase biospanning (SPB) scheme in which the target antigen concentration was decreased in successive rounds of selection (i.e., 100 nM in rounds 1 and 2; 50 nM in rounds 3 and 4). Having previously used this approach, we speculate an apparent affinity of the selected scFvs in the around moderate nanomolar range. The reason behind such expectation relies on the fact that the SPB is capable of controlling target antigen concentration during selection rounds and, therefore, it allows isolation of scFv candidates with predefined affinity. In line with this, Dennis discussed that the binding affinity between target and displayed ligand can be predicted or even predefined by the concentration of target antigen used in SPB.^35^ For example, reducing the concentration of target to nM range during subsequent rounds of selection can lead to the selection of ligands with subnanomolar affinities. Taking into account the ELISA result (Figure 3) and the number of hydrogen bonds formed between the scFvs and VEGF-A, scFvs H1 and E1 showed stronger binding activity, contrary to scFvs D3 and E9.

The isolation of scFvs with apparent and predicted affinity in the subnanomolar range from a moderate diversity and synthetic phage scFv library (Tomlinson I, 3.8 × 10^8^) in the current study is acceptable when compared with the affinity of anti-VEGFR-2 scFv candidates in the 137-6800 nM range, which were isolated from the highly diverse synthetic ETH-2 Gold library (3 × 10^9^ antibody clones) through solid-phase biopanning.^36^ In another study conducted by Böldicke et al.,^37^ an affinity of 3.8 nM was reported for one of the anti-VEGFR-2 scFvs (A7), isolated from a mouse immune library (undergoing in vivo affinity maturation through somatic mutation) and using a solid-phase selection approach. Taken together, these results underscore the capacity of SPB used in this study for the isolation of scFv clones, possibly with high affinity binding in the nanomolar range.

Both experimental and in silico analyses indicated that all selected scFvs recognize conformational but not sequential VEGFR-2 epitope(s). We tried to preserve the intact amino acid sequence-adapted antigen structure without disturbing the antigen conformation during selection and screening steps, which is a common consequence of solid-phase selection and direct antigen coating on ELISA plates.^11,30,38,39^ For this purpose, we exploited the SPB, in which Ni-NTA and streptavidin-based magnetic beads were alternatively used in successive rounds to capture phage scFvs bound to His-tagged and biotinylated VEGFR-2, as well as an indirect target antigen coating through biotin–streptavidin system was used for screening of the target-specific phage clones. Conformation-specific features of the isolated scFvs were demonstrated through binding to the solution form of VEGFR-2 in the ELISA assay with indirect antigen coating (Figure 3) and to the intact and native structure of cell-associated VEGFR-2 in flow cytometry (Figure 4). In addition, a lack of binding activity to the denatured antigen in SDS-PAGE and immunoblotting (data not shown)—such a phenomenon was previously reported.^37^ Intermolecular interactions analysis between VEGFR-2 and scFvs complexes (Figures S5- S8) further confirmed the binding of the scFvs to nonsequential VEGFR-2 amino acid residues.

Taken together, the experimental and docking results demonstrated that the two tested scFvs are capable competitors of VEGF-A that block its binding region in VEGFR-2. We employed two VEGFR-2-specific scFvs, D3 and H1, in the competition ELISA assay, as they displayed the highest and the lowest binding to VEGFR-2 (Figure 3). Notably, based on docking analysis, the given scFvs were categorized into two distinct classes (Figure 7): Class I, exemplified by D3, employs a steric hindrance mechanism by directly occupying the VEGF-A binding site in VEGFR-2 (i.e., the valley region located between D2 and D3 domains of VEGFR-2), and Class II, represented by H1, competes with VEGF-A via binding to the outside but is approximated to the same region on VEGFR-2.

## Conclusions

In summary, we isolated new anti-VEGFR-2 fully human scFvs from a synthetic phage antibody library. Based on the SPB results, we speculate an apparent affinity of around the moderate nanomolar range for the scFvs. The isolated scFvs could recognize the soluble and cell membrane-associated forms of VEGFR-2. Experimental and in silico results indicated that the scFvs could compete with VEGF-A for binding to VEGFR-2. These blocking scFvs may have the potential to inhibit VEGF-induced tumor cell angiogenesis and proliferation, which needs further studies. Additionally, further research is needed to fully understand the potential of these inhibitors and their efficacy in clinical settings.

## Supporting Information Available

Evaluation of the quality of predicted structural models; results obtained from the QMEAN server; ScFvs-VEGFR-2 docking Parameters; structural validation of the final model D3; structural validation of the final model E1; structural validation of the final model E9; structural validation of the final model H1; hydrogen bond network and hydrophobic interactions plot of the D3-VEGFR2 complex; hydrogen bond network and hydrophobic interactions plot of the E1-VEGFR2 complex; hydrogen bond network and hydrophobic interactions plot of the H1-VEGFR2 complex; and hydrophobic interactions plot of the E9-VEGFR2 complex (PDF)
