# Supplementary material for: Isolation and Characterization of the Vascular Endothelial Growth Factor Receptor Targeting ScFv Antibody Fragments Derived from Phage Display Technology
Source: ACS Omega. 2024 May 6;9(20):21964–73. doi: 10.1021/acsomega.3c10158 (PMC11112697; doi:10.1021/acsomega.3c10158)
Supplement: Supplementary file 1 — ao3c10158_si_001.pdf [file ao3c10158_si_001.pdf]

Isolation and Characterization of VEGFR-2 targeting ScFv Antibody Fragments Derived from Phage Display Technology

Hamid Kazemzadeh[1†], Mahsima Bagheri[1†], Maryam Sepehri[1], Elham Ebrahimi[2], Huan Wang[3], Shozeb Haider[3*], Mitra Kheirabadi[2*], Mohammad Reza Tohidkia[1*]

[1] Research Center for Pharmaceutical Nanotechnology, Biomedicine Institute, Tabriz University of Medical Sciences, Tabriz, Iran.

[2] Basic Science Department, Faculty of Biology, Hakim Sabzevari University, Sabzevar, Iran.

[3] School of Pharmacy, University College London, London WC1N 1AX, UK

Table-1S. Evaluation of the quality of predicted structural models

			SAVES Server					
		**QMEAN Server**	Verify3D	ERATT	PROCHECK regions Residues in			
	Model	Z-score	% Of the residues	Overall Quality Factor	Fav.	Add.	Gen.	Dis.
D3	**SWISS MODEL**	**-0.92**	**91.76%**	**88.10**	**90.5 %**	**6.8%**	**1.8 %**	**0.9%**
E1	**SWISS MODEL**	**-1.14**	**91.39**	**91.09**	**90.0**	**7.7**	**1.8**	**0.5**
E9	**SWISS MODEL**	**-0.62**	**89.51**	**88.33**	**91.7**	**6.8**	**1.0**	**0.5**
H1	**SWISS MODEL**	**-1.37**	**88.39**	**90.04**	**91.4**	**6.8**	**0.9**	**0.9**

Table-2S. Results obtained from the QMEAN Server.

ScFv clones	Model	Total QMEAN-score	C_beta interaction energy	All-atom pairwise energy	Solvation energy	Torsion angle energy
**D3**	Before Refinement	-0.92	-0.52	-1.29	-3.45	0.41
	After Refinement	-0.19	-0.41	-0.54	-3.44	1.15
**E1**	Before Refinement	-1.14	-1.16	-1.66	-3.95	0.50
	After Refinement	-0.29	-0.59	-0.72	-3.73	1.26
**E9**	Before Refinement	-0.62	-0.27	-1.55	-3.55	0.74
	After Refinement	0.02	-0.20	-0.73	-3.19	1.25
**H1**	Before Refinement	-1.37	-1.27	-1.50	-3.41	0.08
	After Refinement	-0.25	-1.08	-0.77	-3.40	1.22

Table-3S. ScFvs-VEGFR-2 docking Parameters.

ScFv clones	E1		E9		D3		H1	
**Complex**	**Total Score**	**Interface Score (I_SC)**	**Total Score**	**Interface Score (I_SC)**	**Total Score**	**Interface Score (I_SC)**	**Total Score**	**Interface Score (I_SC)**
**1**	-314.149	-5.912	-318.373	-4.077	-336.228	-3.666	-396.791	-4.138
**2**	-313.857	-5.784	-317.826	-3.983	-335.873	-3.421	-396.754	-3.836
**3**	-313.725	-5.285	-317.457	-3.298	-335.553	-3.388	-396.633	-3.779
**4**	-313.566	-5.194	-317.236	-3.662	-335.536	-4.845	-396.497	-3.539
**5**	-313.564	-5.105	-317.23	-3.957	-335.527	-4.811	-396.339	-3.65
**6**	-313.342	-4.749	-317.226	-3.087	-335.521	-5.036	-396.071	-3.081
**7**	-313.294	-4.677	-317.214	-3.885	-335.391	-4.495	-396.067	-5.588
**8**	-313.2	-4.451	-317.209	-3.988	-335.387	-4.953	-396.015	-3.46
**9**	-313.169	-4.945	-317.152	-2.765	-335.349	-3.589	-395.99	-3.77
**10**	-313.143	-3.945	-317.145	-4.025	-335.314	-4.391	-395.946	-3.577

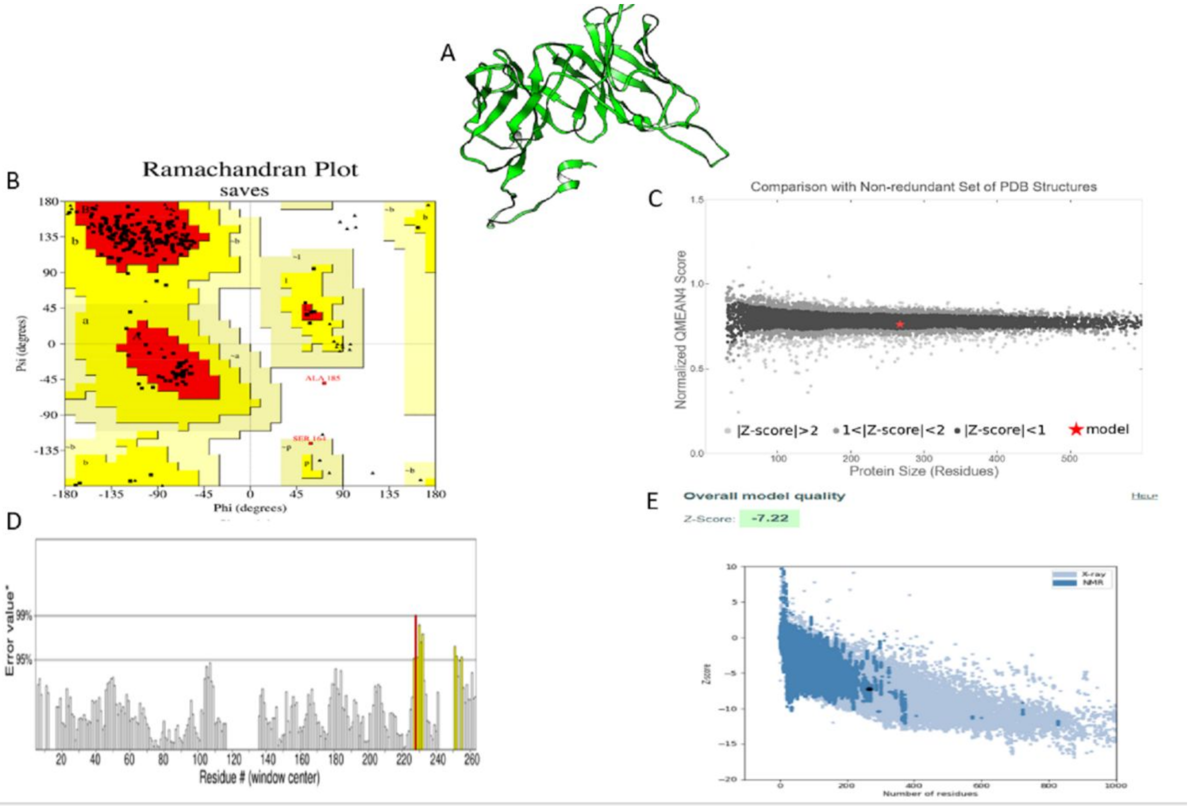

Fig. S1. Structural validation of final model D3. (A) 3D-Structural final with Pymol. (B) Ramachandran plot obtained from Procheck server. The most favored regions, allowed regions, generously allowed regions and disallowed residues (red color) are marked. (C) diagram Comparison QMEAN Z-score with all PDB structures. The model we are looking for is in the form of a red star in the diagram, and the more the model is placed in the black and dark part, the better it will be in terms of quality. (D) The ERRAT plot. (E) The ProSA Z-score. The Z-score indicates overall model quality.

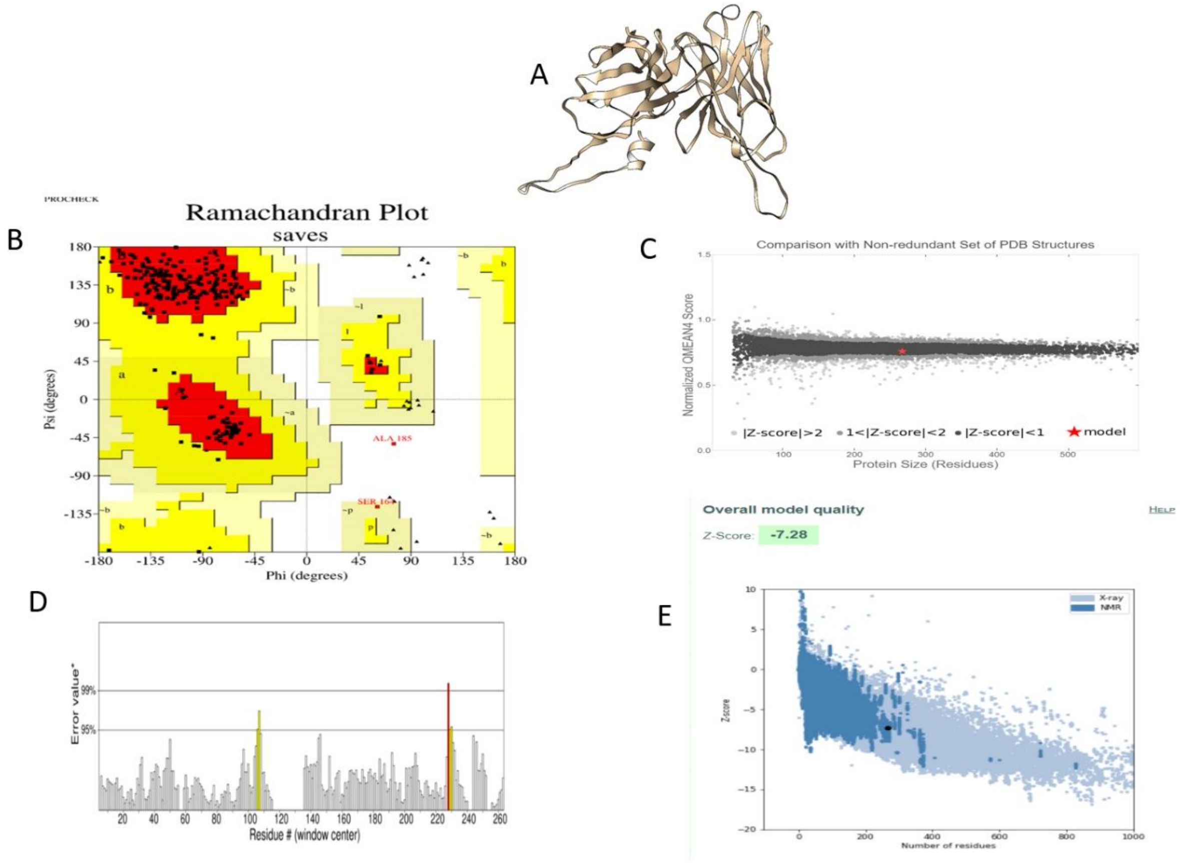

Fig. S2. Structural validation of final model E1. (A) 3D-Structural final with Pymol. (B) Ramachandran plot obtained from Procheck server. (C) diagram Comparison QMEAN Z-score with all PDB structures. (D) The ERRAT plot. (E) The ProSA Z-score. The Z-score indicates overall model quality.

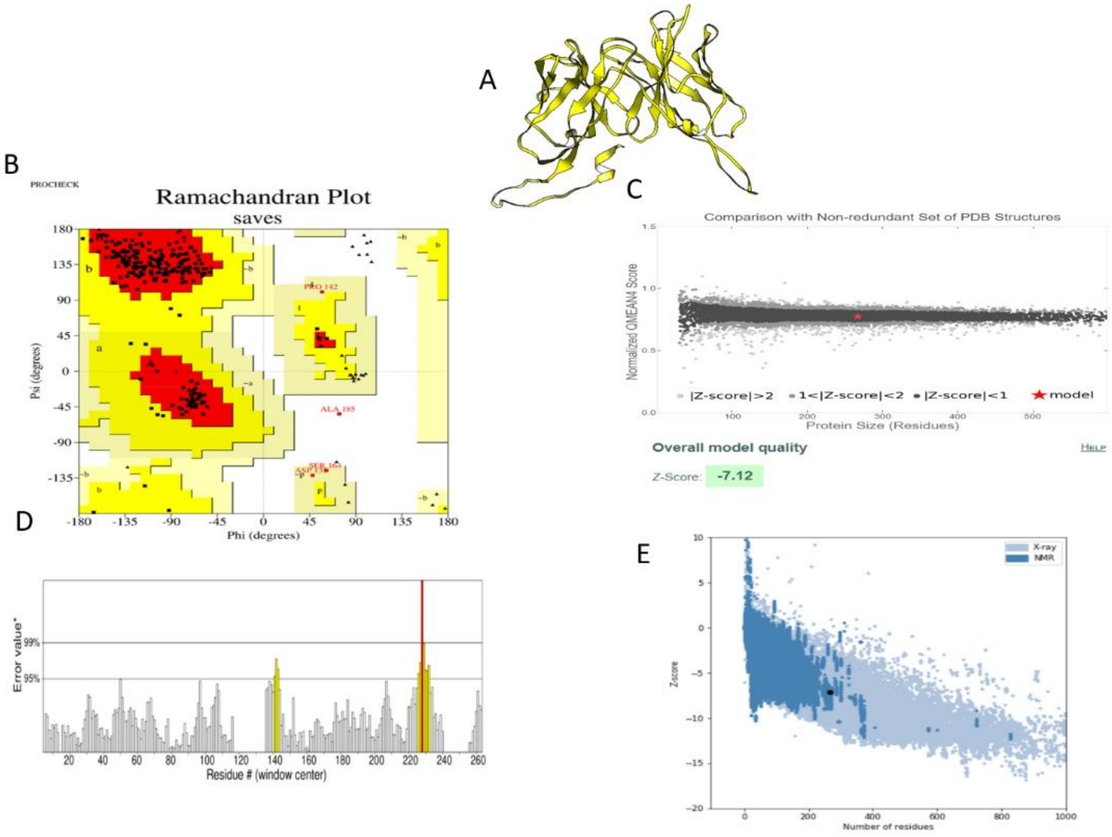

Fig. S3. Structural validation of final model E9. (A) 3D-Structural final with Pymol. (B) Ramachandran plot obtained from Procheck server. (C) diagram Comparison QMEAN Z -Score with all PDB structures. (D) The ERRAT plot. (E) The ProSA Z-score. The Z-score indicates overall model quality.

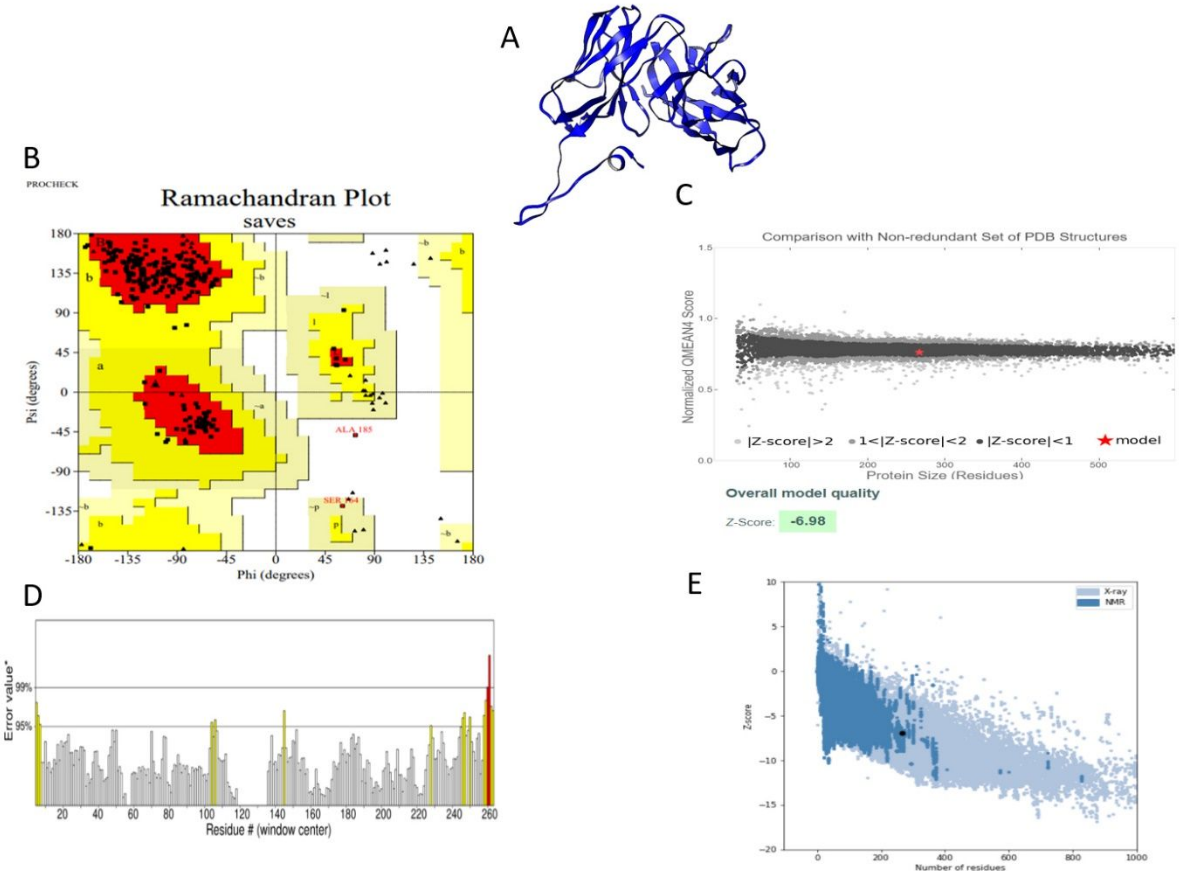

Fig. S4. Structural validation of final model H1. (A) 3D-Structural final with Pymol. (B) Ramachandran plot obtained from Procheck server. (C) diagram Comparison QMEAN Z -score with all PDB structures. (D) The ERRAT plot. (E) The ProSA Z-score. The Z-score indicates overall model quality.

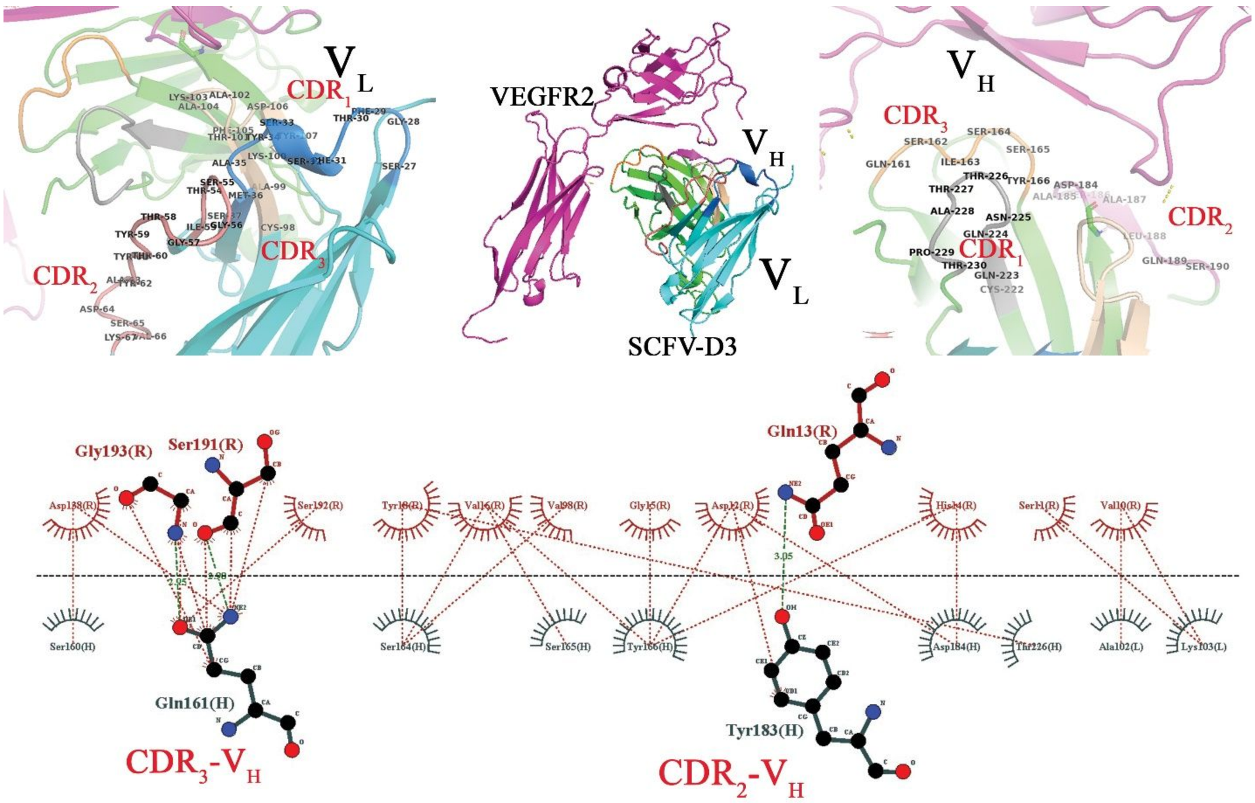

Fig. S5. Hydrogen bond network and hydrophobic interactions plot of D3-VEGFR2 complex.

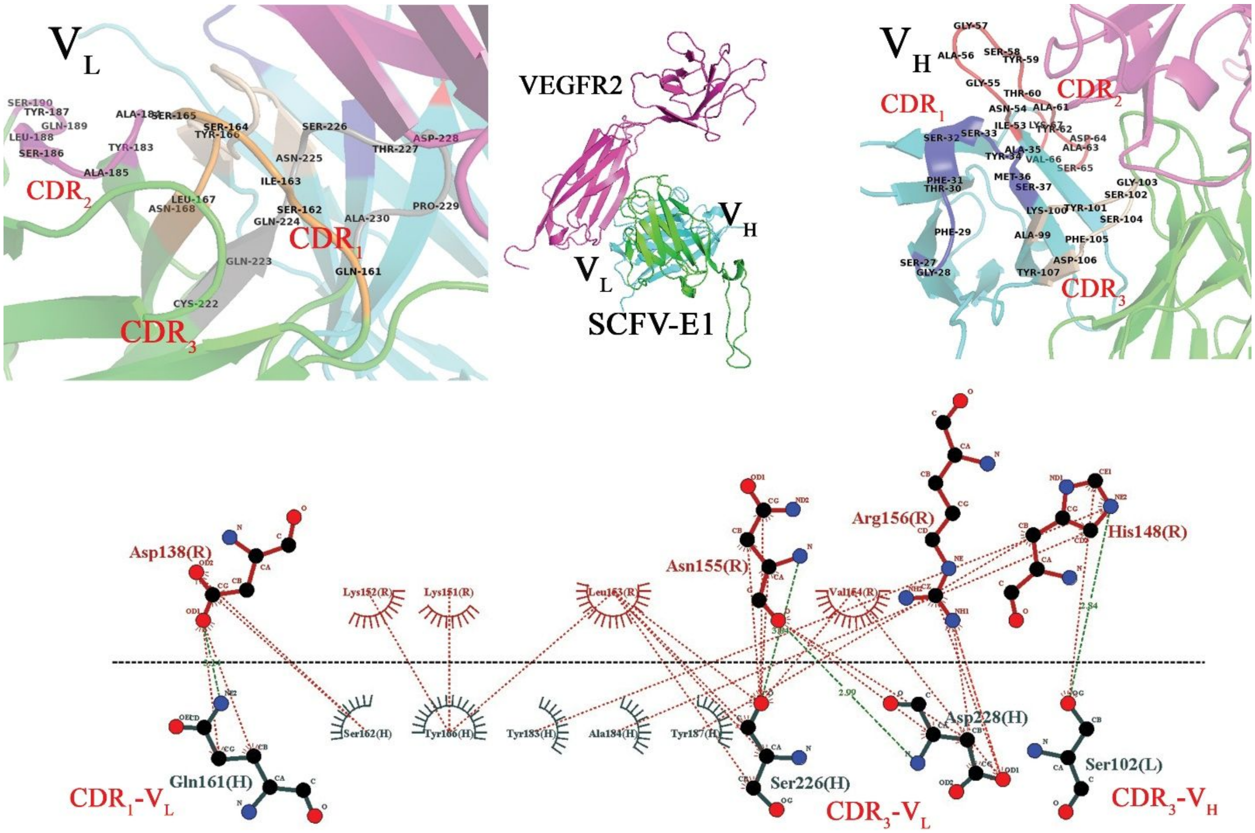

Fig. S6. Hydrogen bond network and hydrophobic interactions plot of E1-VEGFR2 complex.

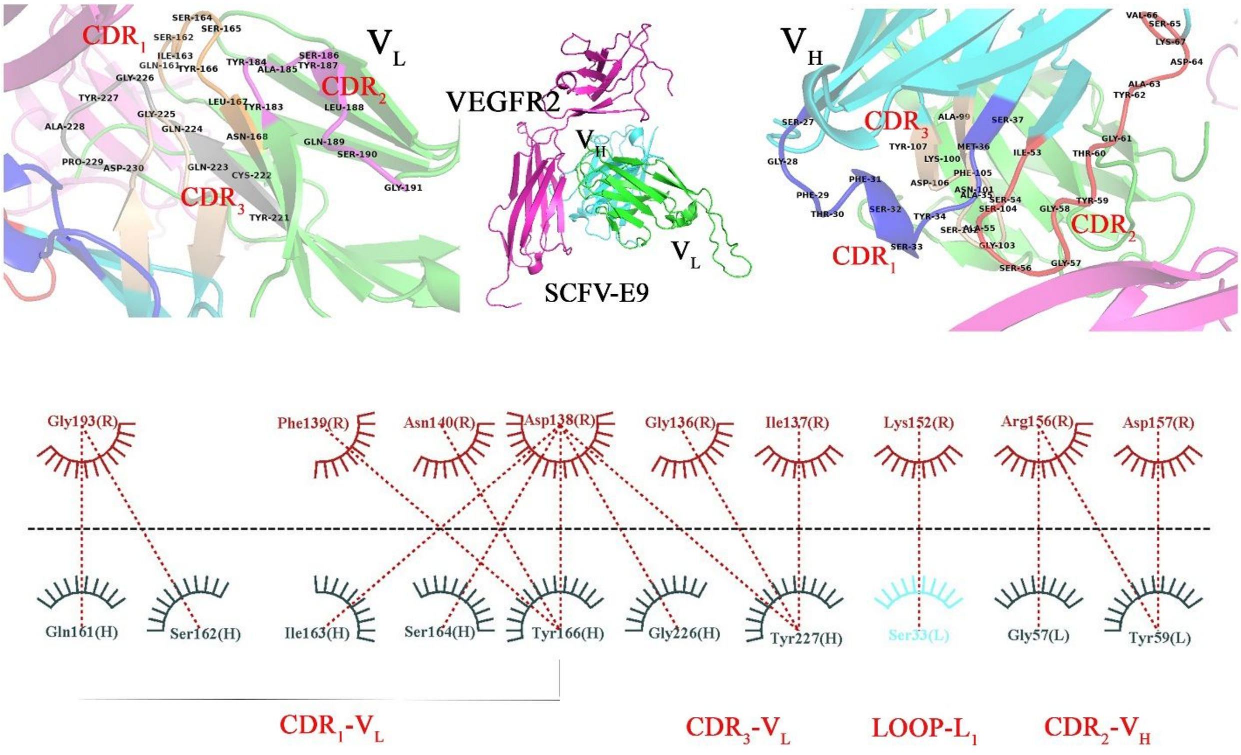

Fig. S7. Hydrophobic interactions plot of E9-VEGFR2 complex.

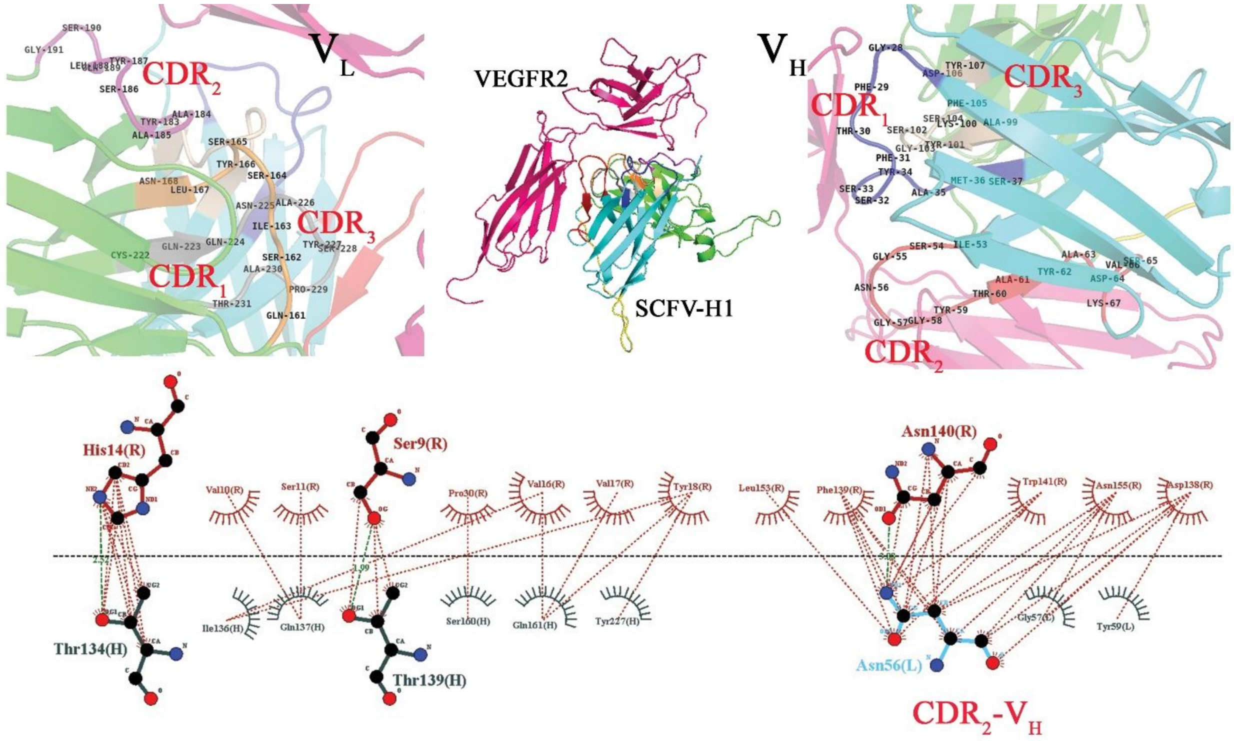

Fig. S8. Hydrogen bonds network and hydrophobic interactions plot of H1-VEGFR2 complex.
